# Autosis as a selective type of cell death

**DOI:** 10.3389/fcell.2023.1164681

**Published:** 2023-04-03

**Authors:** Lingge Bai, Qiong Wu, Xinyue Zhang, Yuting Zhao

**Affiliations:** ^1^ Institute of Future Agriculture, Northwest A&F University, Yangling, China; ^2^ College of Life Sciences, Northwest A&F University, Yangling, China; ^3^ College of Veterinary Medicine, Northwest A&F University, Yangling, China

**Keywords:** autosis, autophagy, autophagic cell death, Na^+^/K^+^-ATPase, Beclin 1, cardiac glycosides, Tat-BECN1 peptide

## Introduction

Autophagy enhances cell survival under many stress conditions, while in certain circumstances, autophagy promotes cell death (Liu and Levine, 2015). In 2013, a new form of cell death was reported by Beth Levine et al., termed “autosis,” which was triggered by prolonged autophagy induction *via* Tat-BECN1 peptide (Shoji-Kawata et al., 2013; He et al., 2022a) or starvation in cultured cells (Liu et al., 2013). Autosis exhibits unique morphological characteristics. At the early stage (Phase 1a), numerous autophagosomes and autolysosomes are formed, mitochondria become electron-dense under the electron microscope, the nuclear membrane is convoluted, and the endoplasmic reticulum (ER) is dilated. At the mid stage (Phase 1b), inner and outer nuclear membranes are separated, and membrane-bound densities are formed. At the late stage (Phase 2), the perinuclear space shows focal ballooning, mitochondria get swollen, and the ER, autophagosomes, and autolysosomes start to disappear. In addition, dying cells become more adherent to the substrate during autosis (Liu et al., 2013; Liu and Levine, 2015). Inhibition of autophagy and Na^+^/K^+^-ATPase genetically or pharmacologically, but not inhibition of apoptosis or necrosis by genetic or pharmacological intervention, blocks autosis (Liu et al., 2013). Autosis also occurs *in vivo*, for instance, in the brain during cerebral hypoxia-ischemia (H/I) (Liu et al., 2013), in the liver during anorexia nervosa (Kheloufi et al., 2015), in the kidney during renal ischemia/reperfusion (I/R) injury (Fernandez et al., 2020), in the heart during cardiac I/R (Nah et al., 2020; Jiang et al., 2022; Nah et al., 2022), and in the skin during terminal differentiation of keratinocyte lineage cells (Koenig et al., 2020). Thus, autosis is a non-apoptotic, non-necrotic, autophagic, and Na^+^/K^+^-ATPase-dependent cell death.

Interestingly, several lines of evidence suggest that autosis is selective, where certain types of cells are more sensitive to physiological or pharmacological autosis inducers and undergo autotic cell death. Here, we discuss the knowns and unknowns about autosis and propose studies to elucidate the molecular mechanisms underlying the selectivity of autosis.

## Selectivity of autosis

**FIGURE 1:**
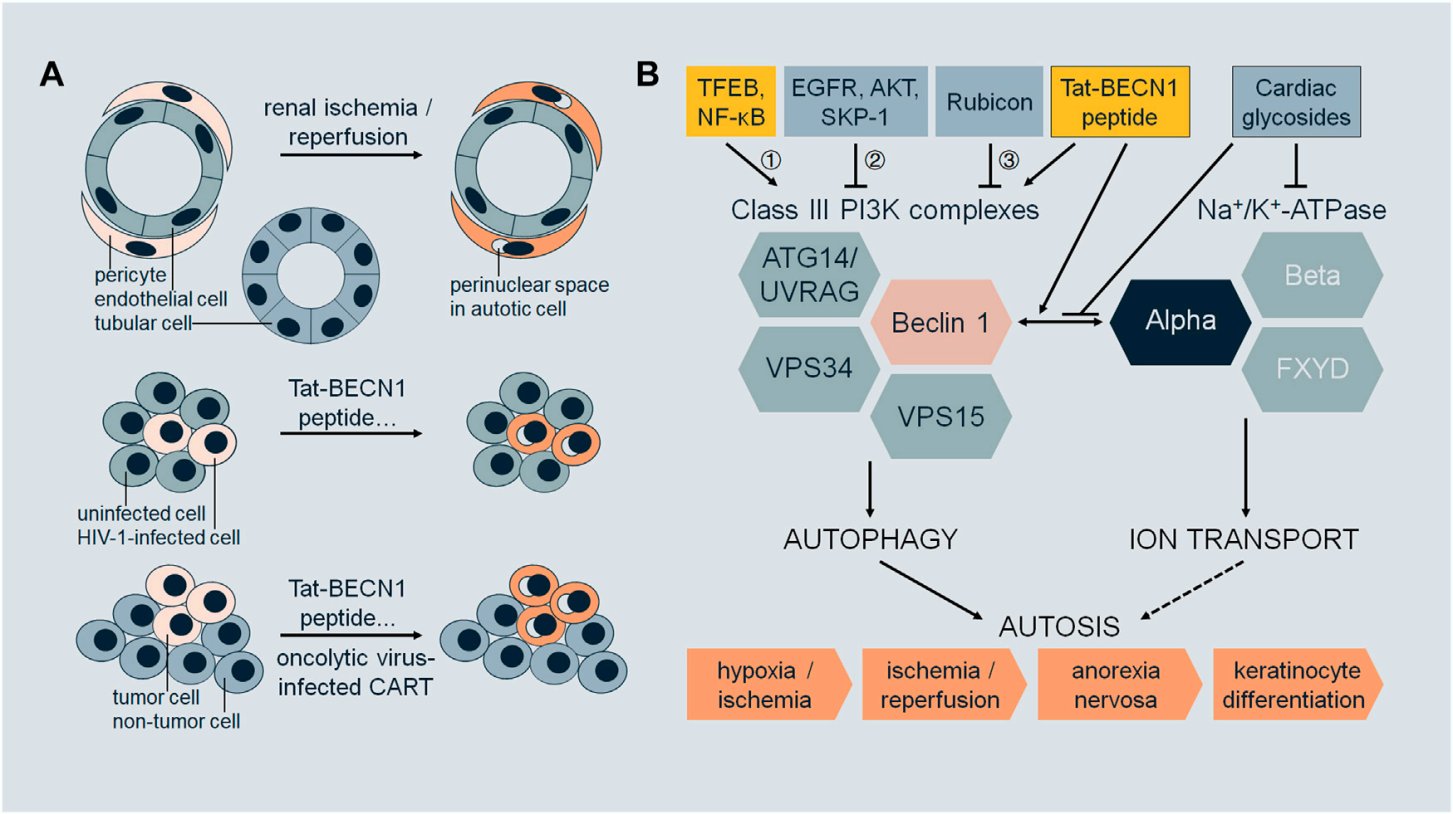
**(A)** Selectivity of autosis. During renal ischemia/reperfusion, pericytes undergo autosis (with morphological features such as focal swelling of the perinuclear space), but not tubular cells or endothelial cells. Upon treatment with Tat-BECN1 peptide or other autosis inducers, HIV-1-infected cells (macrophages or CD4^+^ T cells) undergo autosis, but not uninfected cells. Upon treatment with Tat-BECN1 peptide or other autosis inducers, or oncolytic virus-infected CAR-T cells, tumor cells undergo autosis, but not non-tumor cells. **(B)** Regulation of autosis. Class III PI3K complexes and Na^+^/K^+^-ATPase are two major mediators of autosis, which interact through Beclin 1 and alpha subunits. Pharmacologically, the interaction is enhanced by autosis inducer Tat-BECN1 peptide and blocked by autosis inhibitor cardiac glycosides. Regulatory proteins of autosis act on Class III PI3K complexes by 1) transcriptional regulation, 2) post-translational modifications, and 3) protein–protein interactions. Autosis occurs *in vivo*, in the brain during cerebral hypoxia/ischemia, in the kidney during renal ischemia/reperfusion, in the heart during cardiac ischemia/reperfusion, in the liver during anorexia nervosa, and in the skin during terminal differentiation of keratinocyte lineage cells.

Autosis exhibits cell-type or cell-status specificity (Figure 1A). During renal I/R, autosis occurs in renal pericytes but not in tubular cells or endothelial cells (Fernandez et al., 2020). HIV-1-infected macrophages (Zhang et al., 2018) and CD4^+^ T cells (Zhang et al., 2019) are susceptible to autosis compared to uninfected cells. Autosis induced by oncolytic virus-infected T cells is a potent bystander-killing form for tumor cells but not for non-tumor cells (Zheng et al., 2022). Tat-BECN1 peptide is an autophagy inducer (Shoji-Kawata et al., 2013; He et al., 2022a) and a potent autosis inducer upon high dosage or prolonged treatment (Liu et al., 2013; Zhang et al., 2018; Zhang et al., 2019; Fernandez et al., 2020; Nah et al., 2020; Deng et al., 2022; Nah et al., 2022; Zheng et al., 2022). Tat-BECN1 and derivatives not only induce autosis specifically in HIV-1-infected cells (Zhang et al., 2018; Zhang et al., 2019) but also kill cancer cells in culture *in vitro* and inhibit tumor xenograft growth with negligible systemic toxicity *in vivo* (Wang et al., 2015; Ding et al., 2018; Zhou et al., 2019). These findings imply the therapeutic potential of autosis in infectious diseases and malignancies as it is desirable to eliminate infected cells or cancer cells without affecting normal tissues.

What confers the selectivity of autosis? We speculate that cells with excessive or dysregulated autophagy are likely to undergo autosis. In one scenario, the autophagy levels vary among different cell types. In cells with a higher intrinsic autophagy level, once stimulated with physiological or pharmacological autosis inducers, the autophagy level becomes excessive and fatal. In another scenario, the autophagy machinery has been altered for adaptation in cells which have experienced prior stress, such as viral infection and tumorigenesis. The dysregulation of autophagy in these cells leads to vulnerability upon autophagy-inducing stress that normal cells can tolerate. Moreover, different types of cells may employ specific isoforms and/or regulatory factors of Na^+^/K^+^-ATPase, controlling the progression of autosis. It will be important to compare the molecular features of autosis-prone and reluctant cells in order to illustrate the mechanisms of selectivity.

Notably, the roles of autophagy in infectious diseases and cancers are complex (Mizushima and Levine, 2020; Klionsky et al., 2021). For example, viral pathogens can be removed by autophagy-dependent degradation; however, some viruses, including HIV-1 (Cabrera-Rodriguez et al., 2021) and SARS-CoV-2 (He et al., 2022b), can hijack the autophagy pathway for their replication. Therefore, autophagy can be anti-viral and pro-viral. Autophagy inhibitors chloroquine and hydroxychloroquine have drawn great attention and debate for combating COVID-19, as they show potent preclinical but disappointing clinical efficacy (He et al., 2022b). Depending on the cancer type and stage, autophagy can be anti-cancer and pro-cancer, which should raise concerns when choosing autophagy modulators for clinical studies and use (Galluzzi et al., 2017; Mizushima and Levine, 2020; Klionsky et al., 2021). It is generally thought that autophagy activation may be beneficial to treat diseases that involve defective autophagy, such as neurodegenerative diseases, and autophagy inhibition may be appropriate for cancer therapy when autophagy is upregulated in certain types of cancer. If our speculation that autosis occurs in cells with excessive or dysregulated autophagy is correct, then autophagy activation to a level that induces autosis, instead of autophagy inhibition, may be considered for anti-cancer or anti-viral treatment. On the other hand, for neurodegenerative diseases, autophagy activation to a level that does not induce autosis needs to be achieved to prevent any autotic neuronal loss. It will be interesting to develop reporters and agents to distinguish autophagy activation and autosis induction and investigate the outcomes of autophagy activation with autosis induction or autophagy activation with autosis suppression in disease models.

## Regulators of autosis

So far, studies on autosis mainly focus on the molecular players required for autosis, pathophysiological triggers, and genetic and pharmacological regulators (Figure 1B).

Autosis is autophagy-dependent as downregulation of core autophagy machinery leads to inhibition of autotic cell death, which includes manipulating 1) initiation step, by deletion of ULK1 complex subunit ULK1 (Nah et al., 2020) or ATG13 (Liu et al., 2013); 2) nucleation step, by deletion of Class III PI3K complex subunit Beclin 1 (Liu et al., 2013; Liao et al., 2016; Nah et al., 2020; Deng et al., 2022; Jiang et al., 2022) or ATG14 (Liu et al., 2013), or by administration of inhibitors 3-methyladenine (3-MA) (Liu et al., 2013; Nah et al., 2020) or PIK-III (Zheng et al., 2022); and 3) elongation step, by deletion of ATG5 (Liu et al., 2013; Zhang et al., 2018; Zhang et al., 2019) or ATG7 (Liu et al., 2013; Zhang et al., 2018; Zhang et al., 2019; Koenig et al., 2020; Nah et al., 2020; Nah et al., 2022; Yin et al., 2022). Blocking the maturation of autophagosomes to autolysosomes by bafilomycin A1 (Liu et al., 2013; Zhang et al., 2018; Zhang et al., 2019; Nah et al., 2020) does not attenuate autosis, suggesting that the later step of autophagy is not required.

Regulators of core autophagy machinery are involved in autosis *via* transcriptional regulation, protein–protein interactions, post-translational modifications, or other mechanisms. During the early phase of reperfusion in cardiac I/R, autophagic flux is increased. However, during the late phase of reperfusion (6–24 h in rodent models), autophagic flux is inhibited. Consequently, autosis is prominent in cardiomyocytes during late reperfusion (Nah et al., 2020; Nah et al., 2022). TFEB is a master transcriptional factor for autophagy and lysosomal biogenesis (Palmieri et al., 2011; Settembre et al., 2011). TFEB is upregulated and translocated to nuclei in cardiomyocytes during late reperfusion, and AAV-mediated knockdown of TFEB reduces autosis. Furthermore, TFEB targets such as Beclin 1 and VPS11 are also upregulated during late reperfusion (Nah et al., 2022). Thus, TFEB may regulate autosis by transcriptional regulation. However, it is still unclear which targets of TFEB are indispensable for autosis other than Beclin 1. NF-κB may be another transcriptional factor regulating autosis. Phycocyanin induces autosis in addition to apoptosis in cancer cells (pancreatic), and phycocyanin treatment leads to increased Beclin 1 expression and NF-κB nuclear translocation. NF-κB nuclear translocation inhibitor SN50 reduces Beclin 1 level and autophagy induction caused by phycocyanin (Liao et al., 2016). Rubicon is a well-characterized negative regulator of Class III PI3K complex-2, inhibiting autophagosome maturation and endosomal trafficking (Matsunaga et al., 2009; Zhong et al., 2009; Chang et al., 2019). Rubicon is upregulated during late cardiac I/R. Cardiac tissue-specific knockout of Rubicon normalizes autophagic flux and inhibits autosis during late I/R (Nah et al., 2020). It remains elusive if Rubicon expression during this process is regulated by TFEB or NF-κB, which is also reported to activate autophagy during cardiac I/R injury (Zeng et al., 2013). For cancer cells in culture (including non-small-cell lung cancer and glioblastoma) under hypoxia, autophagy is pro-survival at the early stage and pro-death at the late stage through autosis, when EGFR activity is decreased in the late stage of hypoxia (Chen et al., 2016). Since EGFR phosphorylates and inhibits Beclin 1 (Wei et al., 2013), inhibition of EGFR by tyrosine kinase inhibitor gefitinib or erlotinib promotes autosis in both hypoxia and normoxia (Chen et al., 2016). Oncolytic myxoma virus (MYXV)-infected tumor-specific CAR-T cells (termed CAR-T^10%MYXV^) induce autosis in co-cultured cancer cells (ovarian) (Zheng et al., 2022). The expression level of Class III PI3K complex lipid kinase subunit VPS34 is increased with CAR-T^10%MYXV^ or MYXV treatment. Since SKP-1 mediates ubiquitination and degradation of VPS34 (Xiao et al., 2015) and interacts with MYXV-derived M-T5 protein (Werden et al., 2009), VPS34 level and antitumor activity of CAR-T^10%MYXV^ or MYXV are reduced by overexpression of SKP-1 in cancer cells (Zheng et al., 2022). T-cell-derived IFNγ in the co-culture medium contributes to cancer cell autosis. IFNγ or CAR-T^10%MYXV^ decreases the activity of AKT, which regulates autophagy through phosphorylating Beclin 1 (Wang et al., 2012), while overexpression of constitutively active AKT in cancer cells abolishes the antitumor activity of CAR-T^10%MYXV^ and VPS34 level (Zheng et al., 2022). The ER contact proteins VAPA/B regulate autophagosome biogenesis (Zhao et al., 2018), the knockdown of which attenuates autosis as the ER membrane marker is preserved (Nah et al., 2020). Taken together, autosis activity can be modulated through the expression and/or function of the core autophagy machinery.

Autosis also requires Na^+^/K^+^-ATPase, which is a ubiquitous membrane pump to generate sodium and potassium gradient across the plasma membrane by consuming ATP and is involved in human diseases such as neurological diseases and cancer (Clausen et al., 2017). Na^+^/K^+^-ATPase antagonist cardiac glycosides were identified as autosis inhibitors *via* high-throughput chemical screening (Liu et al., 2013). Several cardiac glycosides have been shown to be protective against autosis, including digoxin (Liu et al., 2013; Chen et al., 2016; Zhang et al., 2018; Zhang et al., 2019; Camuzard et al., 2020; Fernandez et al., 2020; Deng et al., 2022; Yin et al., 2022), neriifolin (*in vivo* during cerebral H/I) (Liu et al., 2013; Fernandez et al., 2020), ouabain (*in vivo* during renal and cardiac I/R) (Fernandez et al., 2020; Nah et al., 2020), digitoxigenin (Liu et al., 2013; Chen et al., 2016), and strophanthidin (Liu et al., 2013). Downregulation of Na^+^/K^+^-ATPase alpha subunit ATP1A1 inhibits autosis in various cell lines (Liu et al., 2013; Zhang et al., 2018; Zhang et al., 2019; Nah et al., 2020; Zheng et al., 2022). ATP1A1 is also the top hit from a genome-wide RNAi screen for autosis inhibitors (Fernandez et al., 2020). HIV-1 infection increases ATP1A1 expression in macrophages and CD4^+^ T memory cells while these infected cells undergo autosis with autophagy-inducing peptides or nanoparticles (Zhang et al., 2018; Zhang et al., 2019). Beclin 1 interacts with alpha subunits of Na^+^/K^+^-ATPase (i.e., ATP1A1, ATP1A2, and ATP1A3), and surprisingly, the interaction occurs on intracellular membranes detected by proximity ligation assays in addition to at the plasma membrane (Fernandez et al., 2020). It is yet to be studied how this interaction regulates autosis, the motifs and residues responsible for the binding, and the subcellular localization of the binding.

Several other autosis mediators are reported, although the molecular mechanisms need further elucidation. Ginger extract induces autosis in cancer cells (pancreatic), likely through AMPK activation and mTOR inhibition (Akimoto et al., 2015). Thioridazine, a dopamine receptor D2 antagonist, induces autotic cell death in cancer stem cells (osteosarcoma) that have increased autophagy activity compared to parental cells (Camuzard et al., 2020). Homocysteine and copper co-treatment induces apoptosis and autosis in cardiomyocytes, which is p62-dependent (Yin et al., 2022). Empagliflozin, a sodium–glucose cotransporter-2 inhibitor, reduces autosis in cardiomyocytes in a Beclin 1-dependent manner (Deng et al., 2022; Jiang et al., 2022).

## Future directions

The molecular mechanisms underlying the selectivity of autosis need further investigation. It is key to understand what makes certain cells (i.e., tumor cells *vs*. non-tumor cells, cancer stem cells *vs*. parental cancer cells, virus-infected cells *vs*. uninfected cells, and renal pericytes *vs*. tubular or endothelial cells) prone to autotic cell death. It may be technically challenging to use multi-omics analysis to find the autosis discrepancies from cells of different origins. A good starting point is to compare the transcriptomes and interactomes among HIV-1-infected and uninfected macrophages or CD4^+^ T cells in culture. In addition to unbiased approaches, the crosstalk between Beclin 1-containing Class III PI3K complexes and Na^+^/K^+^-ATPase could be a focus. Whether HIV-1 infection impacts the expression, activity, and binding of Class III PI3K complexes and Na^+^/K^+^-ATPase shall be explored.

It is important to develop selectivity enhancers or sensitizers of autosis. One strategy is to improve the targeted delivery of known autosis inducers to recipient cells *via* ligand conjugation or nanoparticle packing methods. Another strategy is to conduct chemical and/or genetic screening for positive regulators of autosis induced by stress (i.e., hypoxia or starvation) or Tat-BECN1 peptide. Based on previous studies, the ion transport pathway shall be considered. In addition to Na^+^/K^+^-ATPase, several ion channels such as KCNN4 (calcium-activated potassium channel) and KCNQ2 (voltage-gated potassium channel) show up in the genome-wide RNAi screen for autosis inhibitors (Fernandez et al., 2020). Changes in ion transport or ion channel activity are enriched in Tat-BECN1-, CAR-T^10%MYXV^-, or MYXV-treated cancer cells by RNA sequencing (Zheng et al., 2022). Since focal swelling of the perinuclear space and dilation of ER are unique features of autosis, it would be interesting to investigate if stimulation of ion transport and change of osmolarity in organelles aggravate autosis.

In summary, autosis is a new form of selective cell death with therapeutic potential in malignancies and infectious diseases. Future research on direct molecular reporters of autosis, the crosstalk between autophagy core machinery and ion transport, and the differences between autophagy induction and autosis induction will not only shed light on the molecular basis of autosis but also on the role of autophagy in regulating cell survival and cell death.
