# Corrigendum: Autosis as a selective type of cell death

**DOI:** 10.3389/fcell.2023.1211196

**Published:** 2023-05-09

**Authors:** Lingge Bai, Qiong Wu, Xinyue Zhang, Yuting Zhao

**Affiliations:** ^1^ Institute of Future Agriculture, Northwest A&F University, Yangling, China; ^2^ College of Life Sciences, Northwest A&F University, Yangling, China; ^3^ College of Veterinary Medicine, Northwest A&F University, Yangling, China

**Keywords:** autosis, autophagy, autophagic cell death, Na^+^/K^+^-ATPase, Beclin 1, cardiac glycosides, Tat-BECN1 peptide

In the published article, there was an error regarding the **Affiliations** for Lingge Bai, Qiong Wu and Xinyue Zhang. As well as having affiliation 1, they should also have “2 College of Life Sciences, Northwest A&F University, Yangling, China” for Qiong Wu and Xinyue Zhang, “3 College of Veterinary Medicine, Northwest A&F University, Yangling, China” for Lingge Bai.

In the published article, there was an error in the **Funding** statement. The Funding statement is incomplete. The correct **Funding** statement appears below.

“This work was supported by National Natural Science Foundation of China (32270804), Northwest A&F University Start-up Funding (2190021004), Northwest A&F University Undergraduate Training Programs for Innovation and Entrepreneurship (X202210712422).”

The authors apologize for this error and state that this does not change the scientific conclusions of the article in any way. The original article has been updated.

